# Hyponatraemia After Hip and Knee Replacement: Incidence, Risk Factors, Clinical Consequences and Management in the Era of Enhanced Recovery

**DOI:** 10.3390/clinpract15120236

**Published:** 2025-12-16

**Authors:** Lauren Thornley, James Craig, Thomas W. Wainwright, Robert G. Middleton

**Affiliations:** 1 Orthopaedic Research Institute, Bournemouth University, Bournemouth BH8 8EB, UK; 2 University Hospitals Dorset NHS Foundation Trust, Bournemouth BH7 7DW, UK

**Keywords:** enhanced recovery, hip replacement, hyponatraemia, knee replacement, low sodium, orthopaedics

## Abstract

**Introduction:** Total hip replacements and total knee replacements are among the most frequently performed operations worldwide, and the demand for such procedures is ever-growing. It is essential to focus on preventable medical complications that can arise from these procedures, specifically postoperative hyponatraemia. Postoperative hyponatraemia has an incidence of 20–40% in total hip and knee replacement patient cohorts. Even mild postoperative hyponatraemia is clinically relevant, as it is associated with cognitive impairment and gait disturbance and may undermine the aims of enhanced recovery protocols. Severe postoperative hyponatraemia can lead to seizures, coma, intensive care admission, and death. Although uncommon, the high volume of patients treated in busy orthopaedic centres means such cases will inevitably be encountered. This narrative review summarises the current evidence on incidence, risk factors and consequences of postoperative hyponatraemia in total hip and knee replacement populations. **Methods:** A literature review was performed through the EBSCO and PubMed databases to identify relevant studies. Key search terms included were “hyponatraemia”, “total hip replacement”, and “total knee replacement”. **Results**: The incidence of postoperative hyponatraemia is largely between 20% and 40%; however, there are some outliers to this. Multiple risk factors have been identified through observational studies, including age, preoperative hyponatraemia, female sex and certain medications, which signal a need for a risk stratification strategy that can assist in preoperative assessment and the early identification of patients at higher risk of developing postoperative hyponatraemia. Evidence is scarce regarding interventional studies for the prevention and management of postoperative hyponatraemia, despite multiple studies highlighting the issue. **Conclusion**: Future work should focus on testable, quality improvement interventions, such as automatic sodium checks on postoperative day one, weight-based oral fluid protocols, oral salt supplementation, and escalation pathways for high-risk patients. Incorporating these into enhanced recovery frameworks has the potential not only to optimise safe early discharge for the majority but also to prevent rare but significant complications.

## 1. Introduction

Total hip replacements (THRs) and total knee replacements (TKRs) are amongst the most frequently performed surgeries worldwide, and their numbers are steadily rising. In the United Kingdom (UK) alone, it is projected that both procedures will increase by 40% by 2060, as compared to 2018 [1]. This is primarily driven by an ageing population and growing prevalence of osteoarthritis, and this trend is seen globally, with increasing demands for THR and TKR seen within the United States of America [2,3,4]. Given this ever-growing demand for THR and TKR, attention should continue to focus on the preventable medical and surgical complications that occur. Jørgensen et al. [5] concluded that a relatively small group of patients, identifiable by specific risk factors, account for the majority of potentially preventable medical complications following THR and TKR, highlighting the importance of targeted perioperative strategies.

Within the evolving field of enhanced recovery, same-day discharge has become a key goal. Whilst this is not yet standard practice for all in the UK, it has been successfully adopted for suitable patients in some centres and multiple centres internationally [6]. As the number of day-case procedures is ever growing, there are concerns regarding the incidence of complications compared to inpatient procedures and the delay in which these complications will be recognised. Wainwright et al. [7] highlighted that while day-case procedures are attainable for lower-risk patients, there is room for further research regarding risk stratification models to identify “high-risk” patients for whom a slightly longer hospital stay may be appropriate. Although THR and TKR are highly effective in reducing pain and restoring patient quality of life, the increasing number of patients undergoing these procedures emphasises the importance of safe clinical practice perioperatively to minimise the risk of complications, which may undermine patient recovery.

One critical aspect of perioperative safety is the management of fluid balance and electrolytes, as inappropriate interventions can result in electrolyte disturbances, such as hyponatraemia. Factors such as surgical stress, anaesthesia, and inadequate management of postoperative pain and nausea [8,9,10] can contribute to the development of Syndrome of Inappropriate Antidiuretic Hormone (SIADH), a condition in which the release of antidiuretic hormone is stimulated despite no osmotic trigger, resulting in water retention and dilutional hyponatraemia [9].

Hyponatraemia is defined as a sodium level less than 135mmol/L [11] and is one of the most common electrolyte abnormalities noted in clinical practice [12,13]. The current literature demonstrates that hyponatraemia is a factor for increased mortality risk within general patient populations, with ongoing debate as to whether this is a direct effect or an indicator of illness severity [13,14,15]. Importantly, regarding surgical patients, it has been noted that postoperative hyponatraemia (POH) plays a role in increased mortality and morbidity risk [15,16,17], increased length of stay (LOS) [16,18,19,20,21], increased cost [18] and increased readmission rates [22]. Severe hyponatraemia can manifest as confusion, lethargy and seizures; however, even mild hyponatraemia can result in patients having cognitive impairment, gait disturbance and increased fall risk [11]. Once hyponatraemia is identified in a patient, discharge can be delayed until this stabilises or improves to a safe level. Consequently, it can be appreciated that even mild POH can undermine the aims of enhanced recovery pathways, which are targeted at optimising patient recovery to accelerate the achievement of discharge criteria.

The incidence of hyponatraemia postoperatively in THR and TKR patients is generally between 20 and 40% [19,20]; however, despite this, there has been little attention focused on the prevention and management of postoperative hyponatraemia in orthopaedic patients, particularly within the context of enhanced recovery pathways. It has been reported that patients undergoing THR and TKR are becoming increasingly frail [23] and given the combination of an elderly, comorbid patient population, widespread use of medications known to be predisposed to hyponatraemia, and the evolution of enhanced recovery pathways to day-case procedures, this topic is particularly relevant to current-day practice. We anticipated that POH is common in elective THR and TKR populations and that existing studies would identify consistent risk factors and clinical consequences, along with highlighting gaps in management within enhanced recovery pathways.

## 2. Materials and Methods

An initial scoping review of the available literature established that specific research in this area was limited and too methodologically heterogeneous to perform a formal systematic review. Therefore, a narrative review approach was undertaken for the analysis, underpinned by a rigorous literature search. Studies included in this review were identified through the EBSCO database (including MEDLINE/CINAHL) and PubMed. Key search terms included “hyponatraemia”, “total hip replacement”, and “total knee replacement” to ensure a broad search reach (Table 1).

**Figure 1:**
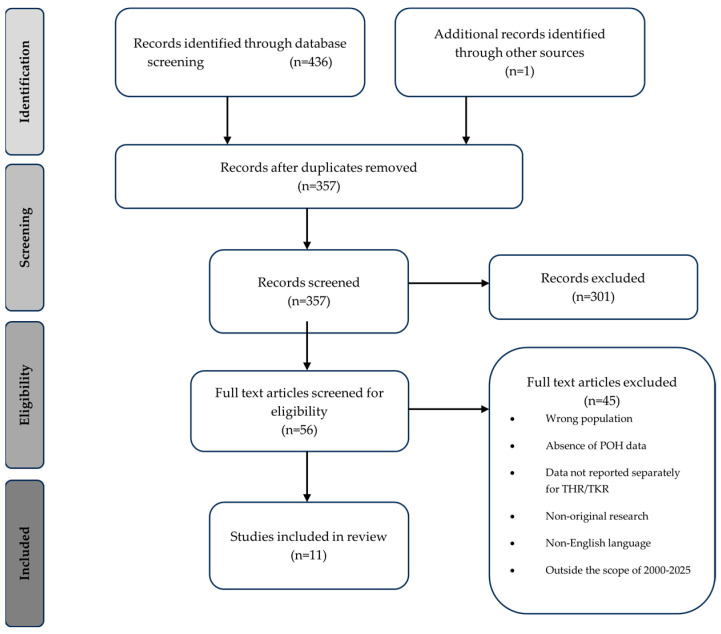
Flow diagram demonstrating the identification and selection of studies included in the narrative review of postoperative hyponatraemia in total hip and knee replacement.

Initial screening of the study titles and abstracts was conducted, followed by an in-depth review of the full text by two separate authors. Studies included were those published between 1 January 2000 and 15 September 2025, with the aim of reviewing data collected from perioperative pathways in keeping with contemporary surgical practice and enhanced recovery pathways. The reference lists from articles found in the literature search were then reviewed to identify any further relevant studies (Figure 1).

Given the small and heterogenous evidence base, all available original research was included, as restricting study types would have excluded clinically relevant information. Studies had to be in English and published between 2000 and 2025. Exclusion criteria are listed in Figure 1. As this was a narrative review PRISMA, full search strings, dual screening, or formal risk-of-bias assessment are not required. This is due to narrative reviews differing fundamentally from systematic reviews in purpose, structure, and methodological requirements and are used when the available evidence is limited or methodologically heterogeneous, which is the case in this topic area [24].

## 3. Discussion

A total of 11 studies met the inclusion criteria (Table 2 and Table 3). The available evidence is limited in quantity and quality, as most studies are retrospective, single-centre cohorts with heterogenous definitions of POH and variable timings and frequency of sodium monitoring. One interventional study was identified, and this was a service evaluation study rather than a controlled trial. Sample sizes ranged from 189 to 3071 patients, and most studies were focused on mixed THR and TKR populations. Biochemical definitions of POH varied, but the majority used a serum sodium of <135 mmol/L. To ensure clarity and that the aims of the narrative review were achieved, the findings were categorised into five themes:

1. Definition and classification of hyponatraemia.
2. Incidence of postoperative hyponatraemia.
3. Risk factors.
4. Clinical consequences.
5. Prevention and management strategies.

### 3.1. Definition and Classification of Hyponatraemia

Hyponatraemia is classed as a sodium (Na) level <135 mmol/L [11]. It is one of the most common electrolyte imbalances encountered in hospitalised patients and can be classified by biochemical severity, fluid status and duration (Table 4):

### 3.2. Incidence of Hyponatraemia in THR and TKR

Whilst hyponatraemia is a common postoperative complication for elective THR and TKR patients with, its reported incidence is variable, and this may be attributed to the methodological heterogeneity across the available studies, including differing thresholds for defining hyponatraemia, variability in the timing of when sodium measurements were taken, and the decision to take sodium measurements routinely or selectively.

In the broader surgical context, POH has been described across a range of surgical specialities, with incidence figures generally reported to be between 15 and 40% [34,35,36]. One study focused on spinal surgery in the elderly population reported an incidence of POH of 15.8%, resulting in an average two-day longer inpatient stay [36]. Neurosurgical and cardiothoracic patients have reported vastly higher rates of 56.6% [35] and 59% [16], respectively. In the neurosurgical cohort, this is secondary to SIADH and cerebral salt-wasting syndrome [37]; meanwhile, in cardiothoracic patients, this contributed to significant perioperative fluid shifts, longer inpatient stays and critical illness [16]. This highlights that POH is a frequent postoperative complication across multiple specialities; however, the patient demographic experienced in elective TKR and THR justifies further evaluation in this review.

Cunningham et al. [20] found an incidence of 21.7% (mild 81.6%, moderate 17.1%, severe 1.4%) in a large retrospective study of 1000 patients, in which serum sodium levels were measured on postoperative day (POD) 1 and POD 2. The standardised measurement of sodium levels optimised the detection of early POH; however, event recognition post-POD 2 may be incomplete and late-onset POH may be underestimated. Furthermore, the retrospective design does not allow for control over confounding factors, such as perioperative medication management and fluid protocols. Cunningham et al. [20] have acknowledged that the missing data for certain variables was a limitation of their study, which may impact the significance of the identified risk factors. Despite these reservations, the incidence rate and severity profile of these cases are consistent with other available literature in the field, supporting the clinical relevance of the studies.

Sah et al. [30] found a higher incidence of 40% (mild: 81.93%, moderate: 14.19%, severe: 3.87%) in a large prospective study, and most commonly identified POH on day 1. The prospective design minimised missing data and allowed for improved control over the exposure definitions. Serum sodium levels were checked daily for inpatients; therefore, the higher incidence may be attributed to this, as late-onset POH may have been captured for those with delayed discharge.

An outlier to these findings is the study by Murkartihal et al. [31], who found an incidence of 84.9%. Several features of this patient cohort should be taken into consideration when understanding this unusually high rate of incidence. This was a single tertiary centre retrospective study based in India, in which patients were admitted to an intensive treatment unit (ITU) postoperatively. This practice does not align with multimodal enhanced recovery pathways, which aim to minimise the surgical stress response and accelerate the achievement of discharge criteria. To illustrate this, discharge was planned for POD 12 once the staples were removed, which is in contrast to the earlier discharges that enhanced recovery pathways facilitate. Prolonged admission suggests recovery for patients was slower, and it may also have resulted in prolonged monitoring of electrolytes and allowed for recognition of cases that would go undetected in patients discharged on the enhanced recovery pathways. For these reasons, the findings of Murkartihal et al. [31] may not be directly comparable to patient cohorts who are managed under enhanced recovery strategies, and their results should be interpreted cautiously within the context of this narrative review.

Despite multiple studies highlighting the impact and consequences of POH, there is only one published study that has attempted to develop an intervention to assist in the management of these patients. Waller et al. [32] performed a closed-loop audit, which demonstrated that the introduction of a direct endocrine referral pathway reduced the incidence of POH after THR and TKR. Furthermore, it was noted that the severity of POH cases that arose from the use of the referral pathway was reduced. Within the first cycle of the audit, 11% of POH patients required ITU admission; however, with the implementation of the referral pathway, no ITU admissions were required.

Although the majority of POH cases following THR and TKR are mild, a small but important proportion progress to severe hyponatraemia (Table 3). Severe cases carry a markedly higher risk of serious outcomes, including the need for intensive care admission, the development of osmotic demyelination syndrome during correction, and death [15,17,32]. While the absolute percentage of patients who develop severe POH is relatively low, the very large numbers of THR and TKR procedures performed each year mean that these complications are encountered in busy orthopaedic units. Therefore, prevention not only focuses on improving recovery and supporting early discharge for the majority of patients but also prevents potentially fatal severe hyponatraemia.

### 3.3. Risk Factors for Postoperative Hyponatraemia

Several risk factors have been identified that can contribute to the development of POH in THR and TKR patients, including patient-related characteristics, medication use, surgical and anaesthetic factors, perioperative fluid management, and enhanced recovery-specific practices (Table 4).

#### 3.3.1. Patient Demographics

Studies have consistently demonstrated that older age is an independent risk factor for developing POH [19,20,21,25,26,29,30,31,32]. It has been observed that there is an age-related reduction in glomerular filtration rate, resulting in the impaired ability to excrete water and increased susceptibility to hypoosmolality and hyponatraemia [38]. It has been repeatedly confirmed that osmoreceptor sensitivity is enhanced in the elderly, triggering increased secretion of antidiuretic hormone (ADH) [39,40]. There is an age-related decrease in the percentage of total body water, as muscle mass reduces with age. This, in turn, can produce greater fluctuations in serum sodium levels, due to the inversely proportional relationship between serum sodium and total body water [38,41]. This mechanism may also explain why patients with a lower body weight are at an increased risk of POH. Female sex has also been identified as a risk factor [30,31], possibly secondary to increased sensitivity to water retention and sodium dilution secondary to increased receptors to ADH in the renal collecting ducts [42]. However, it should be noted that there are studies that have not found sex to be a risk factor [19,20,21,25,27,28,29,32].

#### 3.3.2. Comorbidities and Medications

Several commonly prescribed medications have been linked to increased risk of POH. Most notedly so are thiazide diuretics [19,30,31,32], which impair renal sodium reabsorption and have been linked with hyponatraemia in the older population outside of surgical studies [43,44]. Angiotensin-converting-enzyme inhibitors have been identified as a risk factor [30,31]; it was highlighted that whilst these medications may not present with a hyponatraemia in the preoperative period, they can predispose patients to developing a POH in combination with surgical stress and fluid balance abnormalities. Beta blockers have been associated with POH in elective orthopaedic cohorts [28]; however, the mechanism for this is undefined. It should be highlighted that in general populations, certain beta blockers have been recognised to cause hyponatraemia in the initiation phase of treatment, whilst others do so regardless of their treatment duration [45]. Furthermore, the associations between these cardiovascular medications and the development of hyponatraemia may not be solely due to the medications but also the conditions for which they treat, primarily hypertension and heart failure.

Selective serotonin reuptake inhibitors (SSRIs) and proton pump inhibitors (PPIs) have also been implicated as risk factors for developing hyponatraemia; however, this has not been reflected in an elective orthopaedic cohort. Despite this, one study in traumatic hip fracture patients demonstrated an association between SSRIs and POH [46]; the risk of developing hyponatraemia with SSRIs is associated with initiation of treatment [47]. Cunningham et al. [20] reported that nearly half of the patients who developed POH were taking a PPI in the perioperative period; however, this relationship was statistically insignificant in multivariate analysis. It has been well established in the literature that PPIs can result in patients developing hyponatraemia [46,48,49]. Therefore, despite the statistical insignificance of the relationship noted in the Cunningham et al. [20] study, this is a possible association worth monitoring.

Preoperative hyponatraemia and lower sodium levels have been identified as a risk factor for developing POH in multiple studies [19,20,26,30,31]. The underlying mechanism is not fully defined; therefore, it is hypothesised that increasingly frail or comorbid patients, combined with perioperative factors such as SIADH and fluid shifts, make them more vulnerable to sodium fluctuations and may accelerate the onset and severity of POH [38,41].

#### 3.3.3. Surgical and Anaesthesia

It has been well-established in the literature that the physiological stress of surgery stimulates a secretion of ADH, which may persist for three to five days depending on the amplitude of surgical stress experienced [50]. Anaesthetic procedures may also play a role, as demonstrated by Baker et al. [21], identifying that general anaesthesia was a risk factor for POH. Spinal anaesthesia can trigger sympathetic blockade and vasodilation [51], often requiring additional fluid boluses to maintain blood pressure. In contrast, general anaesthesia amplifies the surgical stress response, with direct effects on ADH secretion and perioperative fluid balance [52]. Volume of blood loss and the requirement for blood transfusion have been identified as risk factors for POH [28,29], likely secondary to haemodilution and stimulation of ADH to replace lost volume.

Waller et al. [32] showed that POH was more common in patients who underwent TKR than THR. The available literature is divided on whether there is a procedure-related risk associated with POH, as certain studies [26,30,31] have demonstrated similar results to those of Waller et al, whereas other studies have found no significant difference between the two procedures [20,21,25,28]. This discrepancy illustrates the importance of further large-scale prospective studies to clarify incidence rates and the risk between THR and TKR. Furthermore, bilateral knee arthroplasty was identified as a risk factor for POH [29,31]. This may be secondary to increased operative time, fluid administration and surgical stress.

#### 3.3.4. Fluid Management

Careful selection of perioperative fluids and monitoring of fluid balance is imperative in mitigating the risk of developing POH. Hypotonic intravenous fluids, such as those containing dextrose, can potentiate hyponatraemia initially triggered by the stress response. Dextrose is rapidly metabolised on infusion and acts as water [53,54]. Therefore, it can be appreciated that in elderly, fragile patients, small changes in total body water can result in significant changes in serum sodium, potentially precipitating or propagating POH.

In contrast, isotonic crystalloids, including 0.9% sodium chloride and balanced solutions, such as Hartmann’s solution, have osmolarities parallel to that of plasma and are designed to maintain intravascular volume without the risk of dilutional hyponatraemia associated with hypotonic solutions [55]. Balanced solutions also contain buffers, which act to maintain normal acid-base balance. 0.9% sodium chloride has similar benefits to Hartmann’s solution; however, it contains an excess of chloride compared to human plasma. As a result, there is a risk of hyperchloraemic metabolic acidosis when administered in large volumes [55].

Perioperative fluid strategy is another priority. Goal-directed fluid therapy is a perioperative strategy in which intravenous fluids are titrated according to haemodynamic parameters to achieve euvolaemia [56,57]. It has been shown in abdominal surgery to reduce complications and accelerate recovery [56]; however, evidence in orthopaedic surgery is limited. Best practice is ill-defined and is generally on a surgeon-by-surgeon basis; however, there is growing evidence that urinary catheters are not required for those undergoing THR and TKR with spinal anaesthetic. This limits the risks of developing a urinary tract infection and optimises postoperative recovery and engagement with rehabilitation. Secondary to this, intraoperative intravenous fluids are minimised to avoid postoperative urinary retention [58]. In conjunction with the enhanced recovery pillar of early oral intake postoperatively, these patients return to drinking early postoperatively, intending to achieve euvolaemia [59].

### 3.4. Clinical Consequences of Hyponatraemia

POH can impact recovery and rehabilitation, neurological function, hospital resource use, and overall patient outcomes. In THR and TKR patients, who are often elderly, frail, and taking multiple medications, even mild hyponatraemia may carry significant clinical implications.

Rehabilitation is particularly affected. Early mobilisation is central to successful recovery after THR and TKR, helping to reduce complications and achieve fast-track discharge. Patients who develop POH may present with weakness, dizziness, or confusion [11], resulting in limited ability to engage in physiotherapy. This delays progress towards discharge and may have knock-on effects on long-term functional outcomes.

An increase in LOS has been consistently demonstrated in patients with POH [21,25,30]. Haider et al. [25] reported a median LOS increase of two days compared with normonatraemic patients, and similar findings have been observed in other retrospective analyses.

The relationship between hyponatraemia, morbidity, and mortality has been reported across surgical and medical populations [15,16,17], although it remains uncertain whether this reflects a causal role or marks frailty and comorbidity. Studies have demonstrated that most cases are mild to moderate (Table 3), yet even these can be clinically significant, especially in older, frailer patients.

Neurological manifestations are perhaps the most immediate concern. Acute hyponatraemia may cause confusion, agitation, impaired concentration, or delirium; in severe cases, seizures, coma, or death can occur [11]. Even mild cases can affect cognition and balance, increasing fall risk during the early mobilisation phase [11]. As early mobilisation is a cornerstone of enhanced recovery protocols, any neurological compromise undermines progress and delays discharge readiness.

### 3.5. Prevention and Management Strategies

The prevention and management of POH in THR and TKR patients requires a collective approach, combining preoperative risk recognition, perioperative fluid optimisation, and alignment with enhanced recovery strategies. Within THR and TKR cohorts, there is a paucity of interventional studies regarding POH. A closed-loop audit in which a direct endocrinology referral pathway was implemented, alongside predefined sodium monitoring, demonstrated a reduction in both the incidence and severity of POH, whilst also eliminating the need to escalate patients to critical care [32]. This demonstrates the potential impacts of interventional studies and highlights the requirement for further prospective studies.

Multiple papers have highlighted the need for a risk stratification model to identify the patients who would be considered at high risk of POH [24,60,61,62]. Currently, there is no validated model available for use in orthopaedic patients; however, use of risk stratification could identify high-risk patients with the potential for preoptimisation before surgery. Preoptimisation could potentially include the involvement of endocrinology to address pre-existing hyponatraemia, reviewing medication and educating at-risk patients on oral fluid intake targets in the immediate postoperative period.

The role of risk stratification needs to be considered further, because the predictive value of identified risk factors is limited, owing to the patient cohort for THR and TKR. These patients are generally older, more likely to be frail and taking multiple medications, so it could be considered that many of these patients would likely be classed as “high-risk”. Thus, standard care for arthroplasty patients should treat all patients as if they are at risk of POH. The only benefit of risk stratification in terms of pathway design is identifying patients who are low-risk, who might not need to adhere to all aspects of standard care, or who might be more suitable for a day case.

### 3.6. Relevance to Enhanced Recovery Pathways

Enhanced recovery pathways are designed to optimise patient recovery, reduce the risk of complications and allow early identification of delayed recovery via evidence-based recommendations. Several key components of enhanced recovery directly intersect with the risk of developing POH and its consequences. In reality, many aspects of orthopaedic enhanced recovery have developed from trial and error, or adopted from other types of surgery, rather than from research. Several key components of enhanced recovery directly relate to the risk of developing POH, and yet there is minimal evidence to support these practices in orthopaedics. For example, the role of intravenous fluid therapy and, in particular, the interplay between the use of intravenous fluids and the need for urinary catheterisation are likely to have a significant bearing on the genesis of POH but lack any evidence base. Whilst early oral intake has been demonstrated to be of benefit following gastrointestinal surgery, the volume of oral fluid intake and the nature of that fluid have not been studied for arthroplasty patients, despite a clear relevance with respect to POH.

Optimised fluid therapy is a pillar of enhanced recovery, ensuring patients achieve euvolaemia; however, inappropriate fluid strategies or administration of hypotonic fluids can precipitate the development of hyponatraemia [55,63]. Multimodal analgesia protocols are aimed at using a combination of analgesics to manage postoperative pain, commonly involving non-steroidal anti-inflammatory drugs, opiates and other adjunctive medications, some of which can promote an SIADH response [64,65]. Postoperative management of nausea and vomiting is another cornerstone of enhanced recovery strategies, as it can improve patients’ ability to return to adequate nutrition, hydration and mobilisation early. Furthermore, it has been associated with patient morbidity and prolonged LOS. Excessive vomiting would result in excess water and sodium loss, possibly potentiating POH. Early oral intake is aimed at restarting oral intake within 24 h postoperatively and may reduce postoperative complications and LOS, whilst aiding in bowel recovery postoperatively [66]. Early patient mobilisation is a critical component of the enhanced recovery pathway and has been shown to reduce the risk of postoperative complications and patient deconditioning [67]. Wainwright et al. [58] advise patients undergoing THR and TKR should “mobilise as early as they are able to in order to facilitate early achievement of discharge criteria”. POH can hinder the aims of the enhanced recovery protocol, as physical manifestations of such a condition include confusion, weakness and increased risk of falls.

Most available studies were conducted in traditional inpatient settings with longer hospital stays, routine postoperative blood tests, and fluid practices that differ from modern enhanced recovery protocols. This may impact the reported incidence of POH, as longer admission and routine daily labs in older cohorts likely increased detection of POH. In contrast, enhanced recovery pathways, with earlier discharge and less routine testing, may underestimate its actual incidence. Furthermore, identified contributors to POH observed in historical inpatient studies, such as prolonged intravenous fluids and delayed oral intake, may be less applicable within enhanced recovery pathways, which emphasise early mobilisation, early drinking, and minimal intravenous fluids.

### 3.7. Gaps in Knowledge and Future Research Directions

Current evidence on POH in THR and TKR cohorts remains limited. Most studies are retrospective and single-centre, with variability in monitoring practices, leading to inconsistent incidence rates and uncertainty around when and how to intervene. Large, multi-centred prospective studies are lacking and would allow further clarification of incidence rates, risk factors and patient outcomes.

There is also no agreed pathway for screening or management. Questions remain over which patients should be monitored, appropriate monitoring protocols, and thresholds for escalation. Preventive measures such as balanced crystalloids, avoidance of hypotonic fluids, and medication review are logical and low cost, but have not been tested together as a structured bundle in orthopaedics. The development of consensus guidelines integrated into enhanced recovery protocols relevant to this population would be highly valuable (Table 5).

Looking forward, digital tools and risk-prediction models may help identify high-risk patients and prompt timely monitoring or referral. This would support consistent practice in a busy and variable ward environment, though there is not yet a validated model within orthopaedics. Finally, there is clear potential for quality improvement projects, for example, testing sequential changes in weight-based fluid and electrolyte management and embedding them into routine enhanced recovery care. These initiatives may aid in standardising the monitoring and management of POH and align with the enhanced recovery principles by reducing preventable complications.

### 3.8. Strengths and Limitations

This review has several strengths, as it focuses on a clinically important but relatively under-valued post-surgical complication in a high-volume surgical population and considers this within the context of modern enhanced recovery pathways. A structured literature search was performed and the data was grouped across incidence, risk factors, consequences and management to ensure all aspects of the topic were covered.

However, it is important to note the limitations of the review. Most of the included studies were retrospective observational cohorts, often single-centre, and primarily conducted within inpatient settings without clearly defined enhanced recovery pathways; therefore, their applicability to the modern concept of fast-track recovery is uncertain. Definitions of POH, timing and frequency of sodium level measurements varied widely between studies, which limited direct comparison and did not allow for meta-analysis. Finally, methodological heterogeneity of the included studies precluded a systematic review being performed. Secondary to this, a formal risk assessment bias was not performed.

## 4. Conclusions

Hyponatraemia is a common and clinically relevant postoperative complication following total hip and knee replacement, yet it remains largely preventable. The prevention and management of POH may be best managed in a multi-disciplinary approach, as it would likely improve consistency in recognition and management; however, implementation may be challenging in busy hospital environments, due to frequent changes in ward staffing and the knock-on effect on adherence to protocols. Given the consistently high incidence reported across elective THR and TKR cohorts, it is reasonable to assume that many patients are inherently at risk of developing postoperative hyponatraemia. Therefore, further evaluation of perioperative pathways needs to take place. One potential approach worth exploring is the design that all patients warrant routine monitoring and prevention strategies, with risk stratification used to identify those who may safely require less intensive surveillance. Despite growing awareness of the risks and consequences of POH, interventional studies in this cohort remain scarce. There is a clear rationale for further quality improvement initiatives focused on structured, standardised fluid management protocols, embedded within enhanced recovery frameworks, to support early discharge while prioritising patient safety. However, these require testing, and definitive recommendations cannot be made until higher-quality evidence exists.

## Figures and Tables

**Table 1:** Search strategy concepts for narrative review of postoperative hyponatraemia in total hip and knee replacements. * is a wildcard to find variations of words starting with hypon.

Concept 1	Concept 2
Hypon * Low sodium Electrolyte imbalance Electrolyte disturbance Combine with **OR**	Total hip replacement Total hip arthroplasty THR Total knee replacement Total knee arthroplasty TKR Joint replacement Arthroplasty Combine with **OR**
Combine with **AND**	

**Table 2:** Summary of studies demonstrating the incidence and severity of postoperative hyponatraemia in total hip and knee replacement.

Author/Year	Population *(n)*	Procedure(s)	Definition of Hyponatraemia	Incidence of POH			
				**Overall**	**Mild**	**Moderate**	**Severe**
Singh (2020) [19]	236	TKR	Na <135 mmol/L + drop ≥5 mmol/L	36.4%	67.4%	23.3%	9.3%
Cunningham (2021) [20]	1000	THR & TKR	Na <135 mmol/L	21.70%	81.6%	17.1%	1.4%
Baker (2023) [21]	3071	THR & TKR	Na <135 mmol/L	12%	Not stated	Not stated	Not stated
Haider (2024) [25]	189	THR & TKR	Na <130 mmol/L	44.40%	24%	57%	19%
Macdonalds (2022) [26]	1000	THR & TKR	Na <135 mmol/L	32.1% 25.7%	87.5% 91%	11.5% 6.6%	0.9% 2.3%
Orfanos (2023) [27]	2721	THR & TKR	Not stated	12.2%	Not stated	Not stated	Not stated
Sinno (2020) [28]	402	THR & TKR	Na <135 mmol/L	26.90%	98.2%	1.8%	0%
Sarkar (2025) [29]	225	THR & TKR	Na <135 mmol/L	30.6%	91.6%	7.2%	1.4%
Sah (2014) [30]	392	THR & TKR	Na <135 mmol/L	40%	81.9%	14.2%	3.9%
Mukartihal (2019) [31]	546	THR & TKR	Na <135 mmol/L	85%	80%	16%	4%
Waller (2025) [32]	Cycle 1–295 Cycle 2–263	THR & TKR	Na <133 mmol/L	Cycle 1–12% Cycle 2–11.7%	41.6% 38.7%	27.8% 51.6%	30.6% 9.7%

**Table 3:** Summary of studies examining risk factors and outcomes of postoperative hyponatraemia in total hip and knee replacement.

Author/Year	Population (*n*)	Procedure(s)	Risk Factors Identified	Outcomes/Complications
Singh (2020) [19]	236	TKR	Older age, lower preoperative sodium, diuretics, total intravenous and oral fluid intake	Reduction in Quality of Recovery-15 score, increased LOS but not clinically meaningful
Cunningham (2021) [20]	1000	THR & TKR	Older age, lower preoperative sodium, and fasting glucose on day 1	No increase in LOS, no increased reattendance or readmission within 90 days, higher rates of inpatient complications
Baker (2023) [21]	3071	THR & TKR	Older age, congestive heart failure, chronic kidney disease, revision, general anaesthesia, higher Charlson Comorbidity Index score, stroke	Increased LOS, greater likelihood of inpatient complications and non-home discharge
Haider (2024) [25]	189	THR & TKR	Older age, increased duration of surgery	LOS increased
Macdonalds (2022) [26]	1000	THR & TKR	Older age, lower preoperative sodium, knee surgery	No impact
Sinno (2020) [28]	402	THR & TKR	Diabetes, blood transfusion, chronic use of beta blockers	No impact
Sarkar (2025) [29]	225	THR & TKR	Diabetes, volume of blood loss >300 mL, older age	No impact
Sah (2014) [30]	392	THR & TKR	Female sex, older age, lower body weight, and lower preoperative sodium, knee replacement, bilateral knee replacement, thiazide diuretics, angiotensin-converting-enzyme inhibitors	LOS increased for knee replacement
Mukartihal (2019) [31]	546	THR & TKR	Preoperative hyponatraemia, female sex, older age, thiazide diuretics, angiotensin-converting-enzyme inhibitors, knee replacement	No impact
Waller (2021) [32]	Cycle 1–295 Cycle 2–263	THR & TKR	Knee replacement, older age, diuretics	Decreased incidence of POH after endocrine pathway introduced with markedly reduced severity, reduced LOS in the second cycle

**Table 4:** Classification of hyponatraemia by biochemical severity, fluid status and duration—associated clinical features and causes are described where appropriate [11,33].

	Category	Criteria	Clinical Features	Common Causes
**Biochemical severity**	Mild	Serum Na^+^ 130–135 mmol/L	Often asymptomatic; may have subtle cognitive or gait disturbance	
	Moderate	Serum Na^+^ 125–129 mmol/L	Nausea, headache, confusion, weakness	
	Severe	Serum Na^+^ <125 mmol/L	Seizures, delirium, coma, risk of death	
**Fluid status**	Hypovolaemia	Loss of total body water and sodium, with sodium loss greater than water	Dry mucous membranes, hypotension, prolonged capillary refill	Vomiting, diarrhoea, diuretic use
	Euvolaemia	Increased total body water with stable sodium content	No oedema, normal blood pressure, normal fluid balance	SIADH, hypothyroidism, Addison’s disease
	Hypervolaemia	Increase in both water and sodium, with water gain exceeding sodium	Peripheral oedema, pulmonary oedema, raised jugular venous pressure	Cardiac failure, renal failure, liver failure
**Duration**	Acute	Onset of <48 h		
	Chronic	Onset of >48 h		

**Table 5:** Practical recommendations for prevention and management of POH in THR and TKR.

Phase	Recommendations	Rationale
**Preoperative**	Identify high-risk patients by identifying risk factors–older age, female sex, preoperative hyponatraemia. Review and adjust high-risk medications where appropriate—antihypertensives, diuretics. Counsel patients on safe oral fluid intake pre/postoperatively.	Early recognition of high-risk patients allows risk stratification and targeted monitoring.
**Intraoperative**	Avoid hypotonic intravenous fluids. Prefer isotonic balanced crystalloids.	Prevents dilutional hyponatraemia, maintains euvolaemia, reduces complications.
**Immediate Postoperative**	Early sodium monitoring (POD 1–2). Optimise analgesia with multimodal strategies while avoiding unnecessary opioids. Proactive antiemetic use to reduce vomiting-related sodium loss.	Detects early POH, limits contributing factors (nausea/vomiting, excessive opioid-related SIADH).
**Ward-based Care**	Encourage early oral intake with isotonic fluids. Avoid excessive free water intake. Monitor fluid balance closely in high-risk patients. Involve endocrinology early for moderate/severe cases.	Supports enhanced recovery goals of early feeding/mobilisation while preventing fluid imbalance.
**Future directions**	Structured peri/postoperative fluid and electrolyte protocols tested within enhanced recovery pathways to assess if incidence and severity of hyponatraemia affected.	Promotes proactive management and early detection of POH.

## Data Availability

No new data were created or analysed in this study. Data sharing is not applicable to this article.

## Abbreviations

The following abbreviations are used in this manuscript:

POH	Postoperative hyponatraemia
THR	Total hip replacement
TKR	Total knee replacement
SIADH	Syndrome of inappropriate antidiuretic hormone
Na	Sodium
POD	Postoperative day
SSRI	Selective serotonin reuptake inhibitors
PPI	Proton pump inhibitors
LOS	Length of stay
ITU	Intensive treatment unit
